# VISUAL DIFFICULTY AND VALUED ACTIVITY AMONG OLDER ADULTS: FINDINGS FROM THE NATIONAL HEALTH AND AGING TRENDS STUDY

**DOI:** 10.1093/geroni/igae098.2534

**Published:** 2024-12-31

**Authors:** Shu Xu, Haowei Wang

**Affiliations:** University of Michigan, Ann Arbor, Michigan, United States; Syracuse University, Syracuse, New York, United States

## Abstract

Vision loss is common among older adults and is associated with reduced activity. However, few studies have examined the value of various activities for older adults with visual difficulty. Using nationally representative data from the National Health and Aging Trends Study 2022, we examined activity preferences and participation among older adults with and without visual difficulty (N=5,807). We explored potential barriers (e.g., transportation, health, and COVID-19) limiting participation in valued activities. Visual difficulty was measured by self-reported vision status and impairments. Valued activities were assessed by four items: visiting friends and family who lived separately, attending religious services, participating in clubs, classes, or other organized activities, and going out for enjoyment. Activities rated as “very important” were considered to be of personal value to the individuals. Weighted sample characteristics showed that 56.3% of participants aged between 65 and 74 years old, and 8.6% experienced visual difficulty: 6.5% near or distance visual difficulty, 1.9% near and distance visual difficulty, and 0.3% blindness. Overall, most older adults (58.7%) rated visiting friends/family as important. Logistic regression results showed that older adults with near and distance visual difficulty were less likely to engage in valued activities (OR = 0.36) and more likely to report health-related (OR = 2.75) or COVID-19 restrictions (OR = 2.04) for ceasing participation in any valued activity, compared to those with no visual difficulty. Findings underscore the significance of developing interventions that bridge the gap between activity preferences and participation for older adults with visual difficulty.

